# Globalists versus nationalists: Bridging the divide through blue marble health

**DOI:** 10.1371/journal.pntd.0007156

**Published:** 2019-07-11

**Authors:** Peter J. Hotez

**Affiliations:** 1 Texas Children’s Hospital Center for Vaccine Development, Departments of Pediatrics and Molecular Virology and Microbiology, National School of Tropical Medicine, Baylor College of Medicine, Houston, Texas, United States of America; 2 Center for Medical Ethics and Health Policy, Baylor College of Medicine, Houston, Texas, United States of America; 3 Department of Biology, Baylor University, Waco, Texas, United States of America; 4 James A. Baker III Institute for Public Policy, Rice University, Houston, Texas, United States of America; 5 Scowcroft Institute of International Affairs, Bush School of Government and Public Service, Texas A&M University, Texas, United States of America; University of Washington, UNITED STATES

With the launch of the United Nations Millennium Development Goals (MDGs) in the year 2000, and later continuing through the 17 Sustainable Development Goals (SDGs) begun in 2016, we have been living in a time of globalism, marked by unprecedented levels of overseas development assistance from wealthy nations, especially the group of seven (G7) nations. A key element of both the MDGs and SDGs is health, with the recognition that poor health represents a major driver of poverty because of its disproportionate impact on economic productivity, child development, and the vitality and security of girls and women. Therefore, perhaps the biggest impact of this 21st century globalism has been the rise of a new era of global health, marked by the creation of new institutions such as the Bill & Melinda Gates Foundation, Gavi, and the Vaccine Alliance and an unprecedented level of financial support to provide access to essential medicines and preventive measures for HIV/AIDS, tuberculosis, and malaria through the Global Fund to Fight AIDS, Tuberculosis, and Malaria; the United States President’s Emergency Plan for AIDS Relief (PEPFAR); and the US President’s Malaria Initiative (PMI). There has also been substantial US government assistance for neglected tropical diseases (NTDs) through the US Agency for International Development (USAID) NTD Program and its United Kingdom counterpart, the Department for International Development (DFID), so that now more than 1 billion people receive treatment annually with a “rapid impact” package of medicines [1, 2].

But as we enter the third decade of this new century, we have also seen the rise of a new nationalism in a number of large and important countries and populations [3, 4]. The most notable example perhaps is the 2016 election of President Donald Trump, whose Make America Great Again initiatives focus on economic protectionism and a transactional foreign policy that emphasizes immediate gains. The US is not alone in its pivot to nationalistic activities and conservatism. The Brazilian government, led by newly elected Jair Bolsonaro, vows to be tough on crime while loosening environmental protections and placing former military leaders in key leadership positions [5]. Similarly, in Europe, we’re seeing new nationalist regimes ascend in Italy and Hungary and in a post-Brexit England, while Steve Bannon, President Trump’s former America First advisor, is regrouping nationalist parties in several countries on the European continent [3]. Globalist–nationalist divides are also deepening across Asia and Africa, in Indonesia, Thailand, and South Africa, and elsewhere [3].

A concern is that these new nationalist trends could curtail or halt the expansions in global health that we have witnessed over the previous 2 decades. Ultimately, there is a fear that retreating from globalism might go hand in hand with abandoning the United Nations Global Goals for health and international development.

However, in a series of articles published in *PLOS Neglected Tropical Disease*, *PLOS Medicine*, and elsewhere [6–10], and later in a single-author book [11], I have highlighted a new global health trend, which could still resonate with nationalist regimes. The concept “blue marble health” refers to my findings that most of the world’s poverty-related neglected diseases, including the NTDs and the “big three” diseases—HIV/AIDS, tuberculosis, and malaria—are in fact most widely prevalent in the group of 20 (G20) economies [6–11]. Specifically, these diseases predominate among the poor living in impoverished areas located near and amid wealth. Such individuals are sometimes also referred to as the “poorest of the rich” [12].

As many of the G20 nations, including the US, Brazil, Indonesia, and the European countries, grow their nationalist movements, it’s worth highlighting the fact that their poorest populations now account for most of the world’s poverty-related neglected diseases. The fact that neglected diseases represent significant drains on national economies and actually have been shown to promote poverty [1] suggests that their control or elimination should become priorities for government leaders and stakeholders. Indeed, one of the most cost-effective means to accelerate G20 economies would be through NTD control and elimination [11]. Because the G20 gross domestic products (GDPs) constitute most of the global economy, neglected disease reductions could become the most straightforward way to promote global economic development.

**Fig 1:**
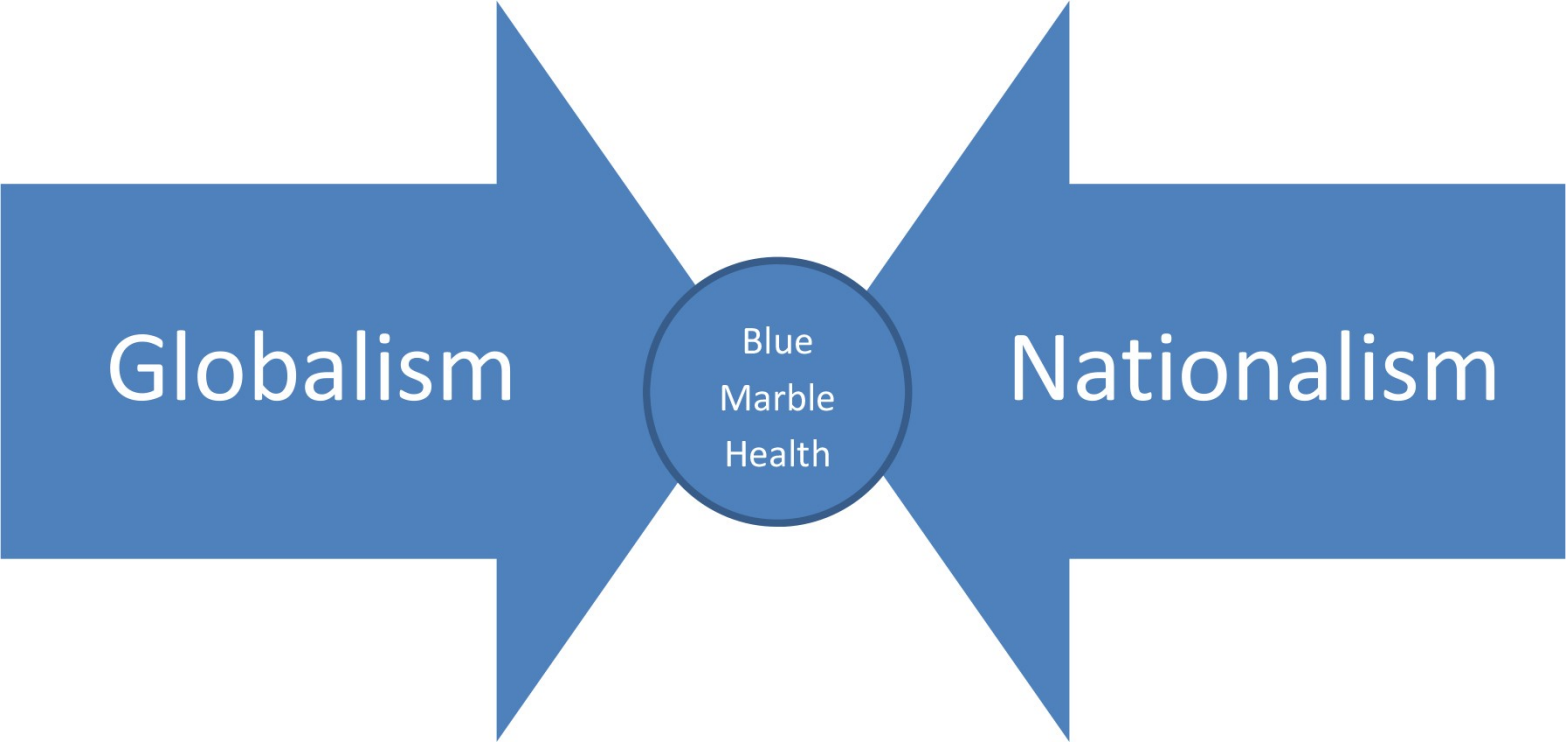
Finding common ground through blue marble health.

Could blue marble health become an important theme to mediate the differences between the globalists and nationalists (Fig 1)?

Clearly, the G20 nations, even under nationalist regimes, might benefit from blue marble health policies. However, this can occur only if nationalism does not drive up disease because of political destabilization, emigration, and loss of potential markets due to negative economic consequences.

It’s also critical that nationalism among the G20 nations does not ignore the rest of the world. What about the remaining deeply impoverished and often conflict-ridden nations at the bottom? The fact that roughly two-thirds of the world’s neglected diseases occur among the G20 [11] must not become an excuse to restrict neglected disease elimination efforts exclusively to the enlightened self-interests of nationalist regimes in a new world order. Control of the NTDs and big three diseases in Africa and the poorest countries of Asia still depends heavily on overseas development assistance through mechanisms of USAID; DFID; PEPFAR; PMI; and The Global Fund to Fight AIDS, Tuberculosis and Malaria (GFATM). Therefore, advancing global health advocacy and policies will need to proceed on two fronts: continuing current assistance activities for the world’s poorest nations while expanding the blue marble health concept among the G20.

G20 outreach and blue marble health also extends to the research and development (R&D) agenda for new drugs, diagnostics, vaccines, and vector control technologies [11]. Currently, the overwhelming global health R&D expenditures arise from the US, UK, and a handful of European nations together with some newer activities through the Japanese Global Health Innovation Technology (GHIT) and Korean Research in Global Health Technology (RIGHT) funds. Accelerating global health R&D expenditures to include the underachievers in this area, such as Brazil, Russia, India, China, and South Africa (the BRICS) is also fundamental to addressing blue marble health.

There are worries that the current nationalist and neoconservative movements could undermine the global health initiatives and outreach that have served us so well since 2000. Blue marble health could become an important health policy framework to mediate the new globalist–nationalist divides.
